# Fluid management in mechanically ventilated children with acute circulatory failure based on the pleth variability index in a pediatric ICU

**DOI:** 10.1186/cc14263

**Published:** 2015-03-16

**Authors:** H Bouguetof, M Negadi, K El Halimi, L Zemour, D Boumendil, Z Mentouri

**Affiliations:** 1 University Ahmed Benbella Oran 1, Oran, Algeria

## Introduction

The pleth variability index (PVI) is a new dynamic index obtained by automatic estimation of respiratory variations in the pulse oximeter waveform amplitude. This noninvasive and continuous hemodynamic monitoring has been recently proposed in mechanically ventilated patients to guide fluid therapy. We recently acquired a PVI monitor in 2014. PVI is calculated by measuring changes in perfusion index (PI) during the respiratory cycle as follows: PVI = ((PImax - Pimin) / PImax) × 100. This study aimed to investigate whether PVI at baseline can predict fluid responsiveness.

## Methods

In our pediatric ICU we started a prospective and observational study. Between January and November 2014, nine mechanically ventilated children were investigated using PVI and transthoracic echocardiography for each patient with acute circulatory failure (ACF): tachycardia, hypotension, oliguria, delayed capillary refilling or hemodynamic instability despite vasopressor drugs. Intervention: standardized volume expansion.

## Results

Significant changes in stroke volume were observed after volume loading (VL) ≥10% in eight patients (responders (R)) and <10% in one patient (nonresponder (NR)). Before VL, PVI was significantly higher in R than NR at baseline ((19.75 ± 3.15%) vs. (9% ± 0.00%), *P *< 0.0001), and decreased significantly in R from baseline to after VL ((19.75% ± 3.15) vs. (12.5% ± 2.828), *P *< 0.0001).

## Conclusion

In this study, PVI seems to predict fluid responsiveness in ventilated children with ACF.

